# Lesser-Known Molecules in Ovarian Carcinogenesis

**DOI:** 10.1155/2015/321740

**Published:** 2015-08-03

**Authors:** Ludmila Lozneanu, Elena Cojocaru, Simona Eliza Giuşcă, Alexandru Cărăuleanu, Irina-Draga Căruntu

**Affiliations:** ^1^Department of Morpho-Functional Sciences, University of Medicine and Pharmacy “Grigore T. Popa”, 16 University Street, 700115 Iaşi, Romania; ^2^Department of Mother and Child Medicine, University of Medicine and Pharmacy “Grigore T. Popa”, 16 University Street, 700115 Iaşi, Romania

## Abstract

Currently, the deciphering of the signaling pathways brings about new advances in the understanding of the pathogenic mechanism of ovarian carcinogenesis, which is based on the interaction of several molecules with different biochemical structure that, consequently, intervene in cell metabolism, through their role as regulators in proliferation, differentiation, and cell death. Given that the ensemble of biomarkers in OC includes more than 50 molecules the interest of the researchers focuses on the possible validation of each one's potential as prognosis markers and/or therapeutic targets. Within this framework, this review presents three protein molecules: ALCAM, c-FLIP, and caveolin, motivated by the perspectives provided through the current limited knowledge on their role in ovarian carcinogenesis and on their potential as prognosis factors. Their structural stability, once altered, triggers the initiation of the sequences characteristic for ovarian carcinogenesis, through their role as modulators for several signaling pathways, contributing to the disruption of cellular junctions, disturbance of pro-/antiapoptotic equilibrium, and alteration of transmission of the signals specific for the molecular pathways. For each molecule, the text is built as follows: (i) general remarks, (ii) structural details, and (iii) particularities in expression, from different tumors to landmarks in ovarian carcinoma.

## 1. Introduction

There are several aspects which place the ovarian cancer in the focus of the scientific community. Its high mortality rate, due to the nonspecific symptoms that determine a delay of early diagnosis, the postsurgical treatment relapses, and the lack of favorable response to chemotherapy for most of the cases [1] require a better understanding of its mechanism and, implicitly, of the molecules that govern its behavior.

Although major progresses have been recorded in recent years in the knowledge of the complex signaling pathways involved in ovarian carcinogenesis [2], the deciphering of its pathogenic journey is far from being complete. The information on the genic and proteomic background of ovarian carcinoma (OC) could be regarded as a giant puzzle which is not yet assembled in order to form the entire image. On the basis of the molecular configuration of the signaling pathways, the interest of the researchers is focused on the identification of those components which could represent either new prognosis markers or new therapeutic targets, or both [3]. The difficulty of this endeavor is augmented by the histologic heterogeneity of ovarian tumors [4].

Even if in the last 15 years over 500 reports on the relationship between the molecular profile and tumor behavior [5, 6] have been available in the mainstream publication, no new prognostic factor is yet confirmed and accepted. The ensemble of potential biomarkers in OC includes more than 50 molecules [5], from which the best known are WT1 and p53 (as oncogenes and tumor suppressor genes), Ki67, PCNA, and topoisomerase II (as proliferation markers), cyclins and their inhibitors (as cell cycle regulators), TRAIL and their receptors, Fas and Fas-L, Bcl-2, Bax, and caspases (as markers of apoptosis), BRCA and PARP-1 (as DNA repair enzymes), CD31, CD34, VEGF, COX-2, and MMPs (as angiogenesis markers), T lymphocytes and their regulatory protein (as immunological factors), EGFR and Her-2 (as tyrosine kinase receptors) and their signaling pathways, and cadherin–beta-catenin complex [6]. Moreover, the review of the literature shows inconsistent data on other promising candidates.

Therefore, we believe the description of ALCAM, c-FLIP, and caveolin is worthwhile, because their expression is fewer investigated in OC, thus supporting their classification in the group of lesser-known molecules involved in ovarian carcinogenesis.

The choice of these three molecules with different functions is supported by our aim to illustrate diverse aspects of the events specific for carcinogenesis: disruption of cellular junctions, disturbance of pro-/antiapoptotic equilibrium, and alteration of transmission of the signals specific for the molecular pathways.

These molecules contribute to normal cell function, but their structural stability, once altered, reveals their competency as modulators that trigger the initiation of the carcinogenic mechanism.

The presentation respects the following sequences for each molecule: initial comments, structural features, and expression and known functions applicable in OC, with a corresponding discussion on the prognosis value.

## 2. ALCAM, Member of Immunoglobulin Superfamily Cell Adhesion Molecules

### 2.1. Starting Point

Cell-cell and cell-matrix interactions assist cellular differentiation and proliferation in both normal and pathologic development. Extensively investigated, the incomplete formation and/or remodeling of cell junctions are regarded as initial steps of the carcinogenic mechanism, while the detachment of cells from primary tumors sets in motion a course that favors invasion and metastasis. A particular attention is granted within this context to the cell adhesion molecules (CAMs), which comprise the families of integrins, cadherins, selectins, and immunoglobulin superfamily (IgSF).

The organ specificity of the molecules belonging to IgSF (generically called Ig-CAMs) was studied in normal status and several malignancies [7–18]. For ovarian tumors, there is little specific information that ascertains the involvement of MCAM [19], L1CAM (CD171), EpCAM [20], IgLON [21], and ALCAM/CD166 (Activated Leukocyte Cell Adhesion Molecule) [22–24]. Strictly referring to ALCAM, besides its role of adhesion molecule, it is also a transductor that modulates a large panel of signaling pathways: MAPK, ERK1/2, and JNK [25].

### 2.2. Structural Features

At first identified and isolated as ligand for CD6 [26] in thymic epithelial cells, ALCAM has been found since then in most fundamental tissues in the human body (except for muscle tissue) and in lymphohematopoietic structures. In physiological circumstances, ALCAM is involved not only in cell adhesion processes, but in neurogenesis, hematopoiesis, and immune responses as well [27]. The adhesion mechanism of ALCAM is both heterophilic (ligand-dependent) and homophilic (ligand-independent, regulated by actin cytoskeleton [28]) and is ensured either by interaction at the N-terminal domain or by* cis* oligomerization on cell surface through C-proximal domain [29].

Following the typical structural pattern of immunoglobulins, ALCAM is a type I transmembrane glycoprotein, with three domains: one extracellular (500 amino acids), one transmembranous (22 amino acids), and one short intracellular, cytoplasmic domain (34 amino acids) [30]. The extracellular domain consists of five N-terminal domains of immunoglobulin type; two are variable and three are constant (V1V2C1C2C3) [30]. The gene which codes ALCAM is located on the long arm of chromosome 3 [26].

### 2.3. ALCAM Expression: From Different Tumors to Landmarks in Ovarian Carcinoma

In tumor pathology, ALCAM expression varies from strong (colon, gastric, and pancreatic cancer) [31–33] to weak (breast cancer) [34], depending on cellular type and on the modified microenvironment.

The value of ALCAM as unfavorable prognosis marker is reported in colon [8], pancreas [33], urinary bladder [35], breast [34, 36], and endometrial [37] tumors, melanoma [38], and other types of malignancies [39], while the association between ALCAM strong expression and a favorable outcome is recorded in prostate cancer [40, 41]. Moreover, ALCAM has also been investigated as marker for evaluation of chemotherapy response in the early stages of breast, cervical [42], pancreas [43], and esophageal cancer [44].

Unfortunately, as far as we know, although there are roughly 150 reports on ALCAM in various types of tumors, only one of these focuses on its value as prognostic factor in OC [23], based on the assessment of one human serous OC cell line and human tissue samples.

The role of ALCAM in ovarian carcinogenesis cannot be understood without knowing its behavior in the normal status. The multiple cell interactions promoted by ALCAM are due to the five extracellular binding domains Ig-like, which explain the membranous expression pattern revealed by immunohistochemistry (IHC). In malignancies, when intercellular adhesion is damaged, with loss of membranous contact, ALCAM expression relocates in cell cytoplasm. In other words, any loss of binding is associated with the internalization of ALCAM [23]. Hence, any event that perturbs the connection between ALCAM and its ligands brings about repercussions on the motility of ovarian tumor cells [23].

**Figure 1:**
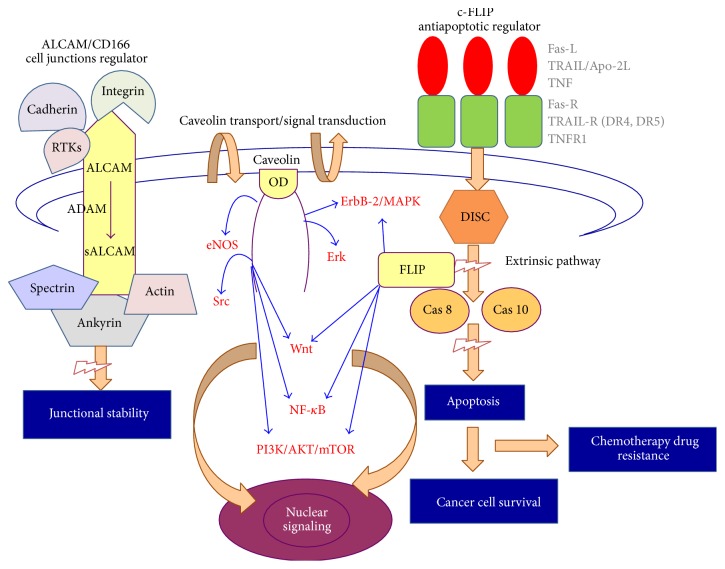
Graphical scheme that illustrates the general structures and action principles for ALCAM, c-FLIP, and caveolin (RTKs: tyrosine kinase receptors, OD: caveolin scaffolding domain, Fas-R/Fas-L, TRAIL/TRAIL-R (DR4, DR5), TNF/TNF-R: death-receptor mediated apoptosis pathway, DISC: Death Inducing Signaling Complex, cas-8: caspase-8, cas-10: caspase-10, and eNOS, Src, ErbB-2/MAPK, Erk, Wnt, NF-*κ*B, and PI3K/AKT/mTOR: signaling pathways).

Thus, it is believed that the membranous expression of ALCAM reflects the maintenance of intercellular stability (Figure 1) and that the cytoplasmic location, resulting from rearrangement of the intercellular junctions, characterizes tumor cells with high potential for invasion and metastasis [23]. This cytoplasmic specificity discriminates the advanced stages from the early ones, which designates ALCAM as a useful marker in the attempt to prove the effect of destruction of the intercellular binding, in tumor* versus* normal context [23]. Consequently, the decrease or absence of ALCAM membrane expression indicates a poor outcome in OC and can be useful in the identification of patients at risk, who need a more frequent follow-up and alternative treatment [23].

However, our experience in the IHC assessment of ALCAM expression in OC (unpublished data) revealed, in a completely unexpected manner, results that contradict the reports in the literature [23]. The membranous pattern of ALCAM, indicator of junction stability and, therefore, of low invasive potential, was predominantly associated with stage III and G3 differentiation. These results assign a higher potential for aggressiveness to the membranous pattern of ALCAM than the one generally recorded in tumor pathology and, particularly, in the ovarian malignancies. This statement opens a series of new perspectives for the reappraisal of the significance of ALCAM expression as indicator for tumor progression ability. In our opinion, a hypothesis worthy of consideration implies the return to the membranous expression, after the cytoplasmic translation, which would reflect a much more aggressive biological behavior than the cytoplasmic profile.

Recent data relying on in vitro (using human epithelial OC cell lines) and in vivo (using human sera and ascites fluid) studies show the existence of a soluble form of ALCAM (sALCAM) [22, 24], which results from its disconnection from the cell membranes (Figure 1). EGFR, in association with other protein molecules (such as phorbol esters and pervanadate),* via* molecular signals triggered in various pathways, ensures the release of ALCAM from ovarian tumor cells through a metalloproteinases-dependent mechanism, regulated by the proteolytic activity of ADAM17/TACE, which determines the occurrence of sALCAM in ascites and serum [22, 24]. Membranous detachment of ALCAM may also occur as result not only of protease degradation but also of methylation of ALCAM promoters [22, 24]. sALCAM conducts tumor growth by coordination of invasion and metastasis [22, 24]. The potential for diffusion in the extracellular liquid recommends the usage of sALCAM as ovarian tumoral biomarker [22, 24], in correlation with the expression level, for sALCAM may be present in the serum of healthy individuals as well [42, 45].

## 3. c-FLIP, A Major Contributor in Mediation of Antiapoptotic Signals

### 3.1. Starting Point

c-FLIP (cellular FLICE-like inhibitory protein) is the main mediator of antiapoptotic events and the negative regulator of the signals monitored by proapoptotic receptors [46, 47], with the involvement of the proteolytic activity of the caspase family members [48, 49] (Figure 1).

c-FLIP is upregulated by several signaling pathways: PI3K/Akt, NF-*κ*B, and MAPK (Figure 1) or downregulated through c-myc, Foxo3a, Fos Jun, or IRF5 pathways [47, 50, 51].

Overexpression of c-FLIP is recorded in various tumors [50, 52–68] and nontumoral diseases (diabetes mellitus, autoimmune syndromes, and multiple sclerosis) [51, 69, 70]. However, as opposed to other apoptotic markers, c-FLIP is less investigated in ovarian tumoral pathology [49, 71–76].

### 3.2. Structural Features

c-FLIP functions as a complex multiprotein system consisting of 3 isoforms with roughly similar structures: a long variant c-FLIP_L_ and two short ones, c-FLIP_S_ and c-FLIP_R_ [47, 77, 78]. The two short variants result from the nucleotide polymorphism in 3′ splice site of c-FLIP gene [51, 79] and are almost equal in size (26 and 24 kDa, resp.) and biochemical arrangement, with only one difference in the C-terminal domain, where c-FLIP_S_ has an addition of 20 amino acids, essential for ubiquitination and proteasomal degradation, which support the antiapoptosis effects [46]. c-FLIP_L_ is the longer variant, weighing 55 kDa, and has a structure similar to caspase-8, which it inhibits and deactivates. The structural analogy between c-FLIP and caspase-8 contributes to unfavorable effects with repercussions in cancer therapy [51]. All three c-FLIP variants display at their N-terminal end two death effector domains (DEDs) [51].

The c-FLIP protumoral effect is achieved by binding c-FLIP to the death receptors through DEDs (in a ligand-dependent or ligand-independent pattern), followed by inhibition of DISC formation by TRAIL and CD95/Fas/APO1 [49] and consequent blockage of the proapoptotic activity of caspase-8 and caspase-10, through inhibition of their activation [51] (Figure 1).

The gene that codes c-FLIP (CFLAR) is located on the 2q33-2q34 chromosome, together with the genes that code caspase-8 and caspase-10 [46, 70]. Any gene alteration afflicts negatively the expression of pro-/antiapoptotic molecules [46, 70].

### 3.3. c-FLIP Expression: From Different Tumors to Landmarks in Ovarian Carcinoma

The overexpression of c-FLIP is reported in experimental studies on cell lines of colorectal carcinoma [52], gastric adenocarcinoma [53], pancreatic [54] and prostate [55] carcinomas, melanoma [56], and tissue specimens corresponding to gastric [57, 58], colorectal [59], gallbladder [60], liver [61], bladder [62], lung [63, 64], and cervix [65] tumors, melanoma [66], Ewing sarcoma [67], and Burkitt lymphoma [68].

In ovarian carcinogenesis, the published data is centered on the antiapoptotic role of c-FLIP in the carcinogenic mechanism by using OC cell lines [49, 71, 72, 76], while only four reports analyze its value as prognosis marker on human tissue samples [71, 73–75].

The presence of c-FLIP is associated with unfavorable prognosis [75], due to its contribution, by regulation of TRAIL signals [73], to the resistance towards the apoptotic receptors [74], which promotes ovarian tumor progression and development of chemoresistance [49] (Figure 1). However, although the knowledge on the various apoptotic receptors and pathways involved in sensitivity or resistance of OC to chemotherapy has increased significantly in the last two decades, this issue is still in permanent upgrade.

Our experience in the IHC appraisal of c-FLIP in OC (unpublished data) reveals, in accordance with the literature [71, 73–75], that the expression of c-FLIP varies significantly between the early and advanced stages, as well as in correlation with the differentiation degree. Our data indicates that a positive expression of c-FLIP characterizes the initial phases of ovarian carcinogenesis, which corresponds to FIGO I stage and differentiation degree G1.

The decrease of c-FLIP expression in advanced stages could be explained either by its interposition only in the initial phases of the apoptosis [74], this process being later inhibited by several other molecules which regulate tumor survival, or it could be possible that the intervention of c-FLIP is no longer necessary for the inhibition of the pathways involved in the maintenance of apoptosis.

It is worth mentioning that, in case of a functional p53, ovarian tumoral cells may escape from the cascade of events specific to apoptosis [71, 75]. Inversely proportional relationships between c-FLIP and p53 are reported, with the c-FLIP increased expression being associated to “wild-type” p53, while mutant p53 is associated to diminished c-FLIP expression [71]. Consequently, the literature describes increased expression of c-FLIP in well-differentiated serous OC and clear cell OC, subtypes which, according to pathogenic classification, are type I tumors, without p53 expression at molecular level [71].

All these data recommend c-FLIP not only as a candidate prognostic factor for OC but also as an useful tool in patients' stratification for innovative treatments which could also take into consideration c-FLIP as therapeutic target [75, 80, 81].

## 4. Caveolin: A Peculiar Mechanotransductor

### 4.1. Starting Point

Caveolins are major structural components of the caveolae [82], located in areas with intense vesicular traffic, where they act as “mechanotransductors” and ensure relay of information towards target molecules [83–85]. Their presence was confirmed in epithelial cells (mainly endothelial cells and pneumocytes), fibroblasts, adipocytes, myocytes [86, 87], and glial cells [88].

They play a dynamic part in the mediation of intercellular and/or extracellular adhesion through cadherins, integrins [89], and fibronectin [90], in the control of endothelial passage, ensuring the stability of the endothelial barrier* via* catenins [91], and inhibit inflammatory processes, through their action on the cytokines [89].

Due to their role as signal transductors, caveolins are involved in various sequences of carcinogenesis [92]. Consequently, several reports in the mainstream publications analyze caveolin in different types of tumors [93–105], with its expression being investigated in OC as well [106–111].

### 4.2. Structural Features

The caveolin is a transmembranous protein with heterooligomeric structure and a molecular weight of 24 kDa. The peculiar form of hairpin is caused by the organization pattern of its five domains: two cytoplasmic N/C-terminals, a C-terminal membrane attachment domain, an oligomerization domain, and a central transmembranous domain [87, 89, 112]. The oligomerized domain comprises a “scaffolding” subdomain (Figure 1), responsible for the interaction between caveolins and various molecules in the vesicular traffic [87, 87, 112].

There are three types described: type 1 with two isoforms (1*α* and 1*β*), type 2, and type 3, all with a molecular weight of 18 to 24 kDa [112]. For caveolins 1 and 3, the role as structural component of caveolae is ascertained, but the function of caveolin 2 remains still undetermined [113].

Caveolins are arranged in a regular pattern, with 100–200 molecules along a caveola, thus forming multiprotein complexes at the submembranous level [114]. Because of the numerous protein and nonprotein signaling molecules at these sites, any structural damage to the caveolae or caveolins generates the inhibition of molecular signaling [115].

### 4.3. Caveolin Expression: From Different Tumors to Landmarks in Ovarian Carcinoma

The profile of caveolins is investigated in various carcinomas, such as breast [96], prostate [97, 98], colon, liver, stomach, esophagus [95, 99], kidney [100], urinary bladder [101], pancreas [102], lung [94], head and neck [103], biliary tree [104], and salivary glands [105], and in sarcoma [93]. The results reveal that their positive expression depends on tumor subtype, grade, or stage and that caveolin inhibition is associated with poor prognosis and metastatic invasion.

Their involvement in the carcinogenic mechanism consists strictly in the regulation of signaling pathways Ras, Raf, ERK, ErbB-2-/MAPK/FAK, Src tyrosine kinase, PI3-K/AKT/mTOR, and NF-*κ*B [89, 93, 116], through their ability to block the activation of the oncogenes v-Src, H-ras, PKA, PKC, and Ras-p42/44 [94, 113], and thus are granted the status of tumor suppressor genes [93, 116] (Figure 1). However, recent evidence shows that caveolins can also act as oncogenes [117–119]. This potential duality, as oncogene* versus* tumor suppressor gene, reflects upon the different molecular pathways, which results in regulation of cell cycle, increase of tumor cell proliferation and invasion potential, promotion of angiogenesis, and the balancing of the apoptotic mechanism [93, 117].

The little existing information regarding caveolins in OC is based rather on experimental researches [83, 106–109] than on human ovarian tissue specimens [110, 111]. The first type of studies, on OC cell lines, shows that the caveolins have the same action mechanism as in the general sequence of carcinogenesis.

The IHC studies on paraffin-embedded samples of normal, benign, and malign ovary reveal that caveolins are present in normal ovarian surface epithelium, in benign pathology, and in early stages of tumor proliferation, with their expression being inhibited as the malignant transformation advances [111]. The prevalent association of the caveolins with the serous subtype is to be noted, in contrast with other OC histologic subtypes [111].

On the other hand, an increased expression of caveolins is ascertained in metastases, as opposed to primary ovarian tumors [110], a fact which suggests that caveolins should not be regarded merely as structural molecules, but also as functional ones, directly involved in the control and regulation of various signals that cross cellular membranes.

In accordance with the literature [110, 111], our results in the assessment of caveolins in OC (unpublished data) indicate that absence of caveolin expression reflects tumor progression, and the correlations with clinicopathological factors and survival variables confirm that its negative expression is associated with a poor prognosis. Extrapolation of IHC results towards the mechanism that governs malignant transformation leads to the idea that in early tumor stages caveolins work as tumor suppressor genes, through the control of junctional contacts, while in advanced tumor stages caveolins function as oncogenes.

Hence, the role of caveolins in the mechanism of ovarian carcinogenesis remains to be clarified, more so taking into account the fact that their behavior varies, according to cellular microenvironment and received signals, from blocking the cellular oncogenic potential to stimulation of tumor growth [111].

## 5. Final Remarks

The current trend in ovarian carcinogenesis is the decoding of the genic and proteomic profile, which would lead to a deeper understanding of the pathogenic mechanism, a clearer explanation for the wide variability in the clinical course, and, also, to the documented validation of molecular markers with prognostic value.

This brief review of the three molecules, ALCAM, c-FLIP, and caveolin, chosen due to the interlocked dialogue they develop in the signaling pathways, is thus fully justified by the perspectives provided through the current limited knowledge on their role in the initiation and progression of ovarian carcinogenesis and on their potential as prognosis factors.

## References

[1] Koshiyama M., Matsumura N., Konishi I. (2014). Recent concepts of ovarian carcinogenesis: type I and type II. *BioMed Research International*.

[2] Dutta D. K., Dutta I. (2013). Origin of ovarian cancer: molecular profiling. *The Journal of Obstetrics and Gynecology of India*.

[3] Ricciardelli C., Oehler M. K. (2009). Diverse molecular pathways in ovarian cancer and their clinical significance. *Maturitas*.

[4] Galic V., Coleman R. L., Herzog T. J. (2013). Unmet needs in ovarian cancer: dividing histologic subtypes to exploit novel targets and pathways. *Current Cancer Drug Targets*.

[5] Le Page C., Huntsman D. G., Provencher D. M., Mes-Masson A.-M. (2010). Predictive and prognostic protein biomarkers in epithelial ovarian cancer: recommendation for future studies. *Cancers*.

[6] Ezzati M., Abdullah A., Shariftabrizi A. (2014). Recent advancements in prognostic factors of epithelial ovarian carcinoma. *International Scholarly Research Notices*.

[7] Ding Y.-B., Chen G.-Y., Xia J.-G., Zang X.-W., Yang H.-Y., Yang L. (2003). Association of VCAM-1 overexpression with oncogenesis, tumor angiogenesis and metastasis of gastric carcinoma. *World Journal of Gastroenterology*.

[8] Weichert W., Knösel T., Bellach J., Dietel M., Kristiansen G. (2004). ALCAM/CD166 is overexpressed in colorectal carcinoma and correlates with shortened patient survival. *Journal of Clinical Pathology*.

[9] Bergom C., Gao C., Newman P. J. (2005). Mechanisms of PECAM-1-mediated cytoprotection and implications for cancer cell survival. *Leukemia & Lymphoma*.

[10] Jezierska A., Matysiak W., Motyl T. (2006). ALCAM/CD166 protects breast cancer cells against apoptosis and autophagy. *Medical Science Monitor*.

[11] Deng C., Zhang D., Shan S., Wu J., Yang H., Yu Y. (2007). Angiogenic effect of intercellular adhesion molecule-1. *Journal of Huazhong University of Science and Technology. Medical Science*.

[12] Roland C. L., Harken A. H., Sarr M. G., Barnett C. C. (2007). ICAM-1 expression determines malignant potential of cancer. *Surgery*.

[13] Siesser P. F., Maness P. F. (2009). L1 cell adhesion molecules as regulators of tumor cell invasiveness. *Cell Adhesion and Migration*.

[14] Park S., DiMaio T. A., Scheef E. A., Sorenson C. M., Sheibani N. (2010). PECAM-1 regulates proangiogenic properties of endothelial cells through modulation of cell-cell and cell-matrix interactions. *The American Journal of Physiology—Cell Physiology*.

[15] Zecchini S., Cavallaro U. (2010). Neural cell adhesion molecule in cancer: expression and mechanisms. *Advances in Experimental Medicine and Biology*.

[16] Campodónico P. B., Joffé E. D. B. D. K., Urtreger A. J. (2010). The neural cell adhesion molecule is involved in the metastatic capacity in a murine model of lung cancer. *Molecular Carcinogenesis*.

[17] Wong C. W., Dye D. E., Coombe D. R. (2012). The role of immunoglobulin superfamily cell adhesion molecules in cancer metastasis. *International Journal of Cell Biology*.

[18] Bombardelli L., Cavallaro U. (2010). Immunoglobulin-like cell adhesion molecules: novel signaling players in epithelial ovarian cancer. *The International Journal of Biochemistry & Cell Biology*.

[19] Aldovini D., Demichelis F., Doglioni C. (2006). M-cam expression as marker of poor prognosis in epithelial ovarian cancer. *International Journal of Cancer*.

[20] Spizzo G., Went P., Dirnhofer S. (2006). Overexpression of epithelial cell adhesion molecule (Ep-CAM) is an independent prognostic marker for reduced survival of patients with epithelial ovarian cancer. *Gynecologic Oncology*.

[21] Sellar G. C., Watt K. P., Rabiasz G. J. (2003). OPCML at 11q25 is epigenetically inactivated and has tumor-suppressor function in epithelial ovarian cancer. *Nature Genetics*.

[22] Rosso O., Piazza T., Bongarzone I. (2007). The ALCAM shedding by the metalloprotease ADAM17/TACE is involved in motility of ovarian carcinoma cells. *Molecular Cancer Research*.

[23] Mezzanzanica D., Fabbi M., Bagnoli M. (2008). Subcellular localization of activated leukocyte cell adhesion molecule is a molecular predictor of survival in ovarian carcinoma patients. *Clinical Cancer Research*.

[24] Carbotti G., Orengo A. M., Mezzanzanica D. (2013). Activated leukocyte cell adhesion molecule soluble form: a potential biomarker of epithelial ovarian cancer is increased in type II tumors. *International Journal of Cancer*.

[25] Ibáñez A., Sarrias M.-R., Farnós M. (2006). Mitogen-activated protein kinase pathway activation by the CD6 lymphocyte surface receptor. *Journal of Immunology*.

[26] Bowen M. A., Patel D. D., Li X. (1995). Cloning, mapping, and characterization of activated leukocyte-cell adhesion molecule (ALCAM), a CD6 ligand. *The Journal of Experimental Medicine*.

[27] Swart G. W. M. (2002). Activated leukocyte cell adhesion molecule (CD166/ALCAM): developmental and mechanistic aspects of cell clustering and cell migration. *European Journal of Cell Biology*.

[28] Nelissen J. M. D. T., Peters I. M., de Grooth B. G., van Kooyk Y., Figdor C. G. (2000). Dynamic regulation of activated leukocyte cell adhesion molecule-mediated homotypic cell adhesion through the actin cytoskeleton. *Molecular Biology of the Cell*.

[29] Van Kilsdonk J. W. J., Takahashi N., Weidle U. (2012). Modulation of activated leukocyte cell adhesion molecule-mediated invasion triggers an innate immune gene response in melanoma. *The Journal of Investigative Dermatology*.

[30] Weidle U. H., Eggle D., Klostermann S., Swart G. W. M. (2010). ALCAM/CD166: cancer-related issues. *Cancer Genomics & Proteomics*.

[31] Tachezy M., Zander H., Gebauer F. (2012). Activated leukocyte cell adhesion molecule (CD166)—its prognostic power for colorectal cancer patients. *The Journal of Surgical Research*.

[32] Ye M., Du Y.-L., Nie Y.-Q., Zhou Z.-W., Cao J., Li Y.-F. (2015). Overexpression of activated leukocute cell adhesion molecule in gastric cancer is associated with advanced stages and poor prognosis and miR-9 deregulation. *Molecular Medicine Reports*.

[33] Kahlert C., Weber H., Mogler C. (2009). Increased expression of ALCAMCD166 in pancreatic cancer is an independent prognostic marker for poor survival and early tumour relapse. *British Journal of Cancer*.

[34] King J. A., Ofori-Acquah S. F., Stevens T., Al-Mehdi A.-B., Fodstad O., Jiang W. G. (2004). Activated leukocyte cell adhesion molecule in breast cancer: prognostic indicator. *Breast Cancer Research*.

[35] Tomita K., Van Bokhoven A., Jansen C. F. J. (2003). ctivated leukocyte cell adhesion molecule (ALCAM) expression is associated with a poor prognosis for bladder cancer patients. *UroOncology*.

[36] King J. A., Tan F., Mbeunkui F. (2010). Mechanisms of transcriptional regulation and prognostic significance of activated leukocyte cell adhesion molecule in cancer. *Molecular Cancer*.

[37] Liang S., Huang C., Jia S., Wang B. (2011). Activated leukocyte cell adhesion molecule expression is up-regulated in the development of endometrioid carcinoma. *International Journal of Gynecological Cancer*.

[38] van Kempen L. C. L. T., Nelissen J. M. D. T., Degen W. G. J. (2001). Molecular basis for the homophilic activated leukocyte cell adhesion molecule (ALCAM)-ALCAM interaction. *The Journal of Biological Chemistry*.

[39] Ofori-Acquah S. F., King J. A. (2008). Activated leukocyte cell adhesion molecule: a new paradox in cancer. *Translational Research*.

[40] Kristiansen G., Pilarsky C., Wissmann C. (2005). Expression profiling of microdissected matched prostate cancer samples reveals CD166/MEMD and CD24 as new prognostic markers for patient survival. *The Journal of Pathology*.

[41] Minner S., Kraetzig F., Tachezy M. (2011). Low activated leukocyte cell adhesion molecule expression is associated with advanced tumor stage and early prostate-specific antigen relapse in prostate cancer. *Human Pathology*.

[42] Ihnen M., Kress K., Kersten J. F. (2012). Relevance of activated leukocyte cell adhesion molecule (ALCAM) in tumor tissue and sera of cervical cancer patients. *BMC Cancer*.

[43] Hong X., Michalski C. W., Kong B. (2010). ALCAM is associated with chemoresistance and tumor cell adhesion in pancreatic cancer. *Journal of Surgical Oncology*.

[44] Tachezy M., Effenberger K., Zander H. (2012). ALCAM (CD166) expression and serum levels are markers for poor survival of esophageal cancer patients. *International Journal of Cancer*.

[45] Faça V. M., Hanash S. M. (2009). In-depth proteomics to define the cell surface and secretome of ovarian cancer cells and processes of protein shedding. *Cancer Research*.

[46] Bagnoli M., Canevari S., Mezzanzanica D. (2010). Cellular FLICE-inhibitory protein (c-FLIP) signalling: a key regulator of receptor-mediated apoptosis in physiologic context and in cancer. *The International Journal of Biochemistry & Cell Biology*.

[47] Öztürk S., Schleich K., Lavrik I. N. (2012). Cellular FLICE-like inhibitory proteins (c-FLIPs): fine-tuners of life and death decisions. *Experimental Cell Research*.

[48] Duiker E. W., van der Zee A. G. J., de Graeff P. (2010). The extrinsic apoptosis pathway and its prognostic impact in ovarian cancer. *Gynecologic Oncology*.

[49] El-Gazzar A., Wittinger M., Perco P. (2010). The role of c-FLIPL in ovarian cancer: chaperoning tumor cells from immunosurveillance and increasing their invasive potential. *Gynecologic Oncology*.

[50] Shirley S., Micheau O. (2013). Targeting c-FLIP in cancer. *Cancer Letters*.

[51] Safa A. R. (2012). c-FLIP, a master anti-apoptotic regulator. *Experimental Oncology*.

[52] Hernandez A., Wang Q. D., Schwartz S. A., Evers B. M. (2001). Sensitization of human colon cancer cells to TRAIL-mediated apoptosis. *Journal of Gastrointestinal Surgery*.

[53] Nam S. Y., Jung G.-A., Hur G.-C. (2003). Upregulation of FLIPs by Akt, a possible inhibition mechanism of TRAIL-induced apoptosis in human gastric cancers. *Cancer Science*.

[54] Elnemr A., Ohta T., Yachie A. (2001). Human pancreatic cancer cells disable function of Fas receptors at several levels in Fas signal transduction pathway. *International Journal of Oncology*.

[55] Zhang X., Jin T.-G., Yang H., Dewolf W. C., Khosravi-Far R., Olumi A. F. (2004). Persistent c-FLIP(L) expression is necessary and sufficient to maintain resistance to tumor necrosis factor-related apoptosis-inducing ligand-mediated apoptosis in prostate cancer. *Cancer Research*.

[56] Griffith T. S., Chin W. A., Jackson G. C., Lynch D. H., Kubin M. Z. (1998). Intracellular regulation of TRAIL-induced apoptosis in human melanoma cells. *The Journal of Immunology*.

[57] Lee S. H., Kim H. S., Kim S. Y. (2003). Increased expression of FLIP, an inhibitor of Fas-mediated apoptosis, in stomach cancer. *APMIS*.

[58] Zhou X.-D., Yu J.-P., Liu J., Luo H.-S., Chen H.-X., Yu H.-G. (2004). Overexpression of cellular FLICE-inhibitory protein (FLIP) in gastric adenocarcinoma. *Clinical Science*.

[59] Ullenhag G. J., Mukherjee A., Watson N. F. S., Al-Attar A. H., Scholefield J. H., Durrant L. G. (2007). Overexpression of FLIPL is an independent marker of poor prognosis in colorectal cancer patients. *Clinical Cancer Research*.

[60] Zong H., Yin B., Chen J., Ma B., Cai D., He X. (2009). Over-expression of c-FLIP confers the resistance to TRAIL-induced apoptosis on gallbladder carcinoma. *The Tohoku Journal of Experimental Medicine*.

[61] Okano H., Shiraki K., Inoue H. (2003). Cellular FLICE/caspase-8-inhibitory protein as a principal regulator of cell death and survival in human hepatocellular carcinoma. *Laboratory Investigation*.

[62] Korkolopoulou P., Goudopoulou A., Voutsinas G. (2004). c-FLIP expression in bladder urothelial carcinomas: its role in resistance to Fas-mediated apoptosis and clinicopathologic correlations. *Urology*.

[63] Salon C., Eymin B., Micheau O. (2006). E2F1 induces apoptosis and sensitizes human lung adenocarcinoma cells to death-receptor-mediated apoptosis through specific downregulation of c-FLIPshort. *Cell Death and Differentiation*.

[64] Wilson N. S., Dixit V., Ashkenazi A. (2009). Death receptor signal transducers: nodes of coordination in immune signaling networks. *Nature Immunology*.

[65] Wang W., Wang S., Song X. (2007). The relationship between c-FLIP expression and human papillomavirus E2 gene disruption in cervical carcinogenesis. *Gynecologic Oncology*.

[66] Bullani R. R., Huard B., Viard-Leveugle I. (2001). Selective expression of FLIP in malignant melanocytic skin lesions. *The Journal of Investigative Dermatology*.

[67] de Hooge A. S. K., Berghuis D., Santos S. J. (2007). Expression of cellular FLICE inhibitory protein, caspase-8, and protease inhibitor-9 in Ewing sarcoma and implications for susceptibility to cytotoxic pathways. *Clinical Cancer Research*.

[68] Valnet-Rabier M.-B., Challier B., Thiebault S. (2005). c-flip protein expression in Burkitt's lymphomas is associated with a poor clinical outcome. *British Journal of Haematology*.

[69] Micheau O. (2003). Cellular FLICE-inhibitory protein: an attractive therapeutic target?. *Expert Opinion on Therapeutic Targets*.

[70] Safa A. R., Day T. W., Wu C.-H. (2008). Cellular FLICE-like inhibitory protein (C-FLIP): a novel target for cancer therapy. *Current Cancer Drug Targets*.

[71] Mezzanzanica D., Balladore E., Turatti F. (2004). CD95-mediated apoptosis is impaired at receptor level by cellular FLICE-inhibitory protein (long form) in wild-type p53 human ovarian carcinoma. *Clinical Cancer Research*.

[72] Lane D., Cartier A., L'Espérance S., Côté M., Rancourt C., Piché A. (2004). Differential induction of apoptosis by tumor necrosis factor-related apoptosis-inducing ligand in human ovarian carcinoma cells. *Gynecologic Oncology*.

[73] Horak P., Pils D., Roessler M. (2005). Common death receptor 4 (DR4) polymorphisms do not predispose to ovarian cancer. *Gynecologic Oncology*.

[74] Ouellet V., Le Page C., Madore J. (2007). An apoptotic molecular network identified by microarray: on the TRAIL to new insights in epithelial ovarian cancer. *Cancer*.

[75] Bagnoli M., Ambrogi F., Pilotti S. (2009). c-FLIPL expression defines two ovarian cancer patient subsets and is a prognostic factor of adverse outcome. *Endocrine-Related Cancer*.

[76] Dong H. P., Ree Rosnes A. K., Bock A. J. (2011). Flow cytometric measurement of cellular FLICE-inhibitory protein (c-FLIP) in ovarian carcinoma effusions. *Cytopathology*.

[77] Irmler M., Thome M., Hahne M. (1997). Inhibition of death receptor signals by cellular FLIP. *Nature*.

[78] Safa A. R., Pollok K. E. (2011). Targeting the anti-apoptotic protein c-FLIP for cancer therapy. *Cancers*.

[79] Ueffing N., Singh K. K., Christians A. (2009). A single nucleotide polymorphism determines protein isoform production of the human c-FLIP protein. *Blood*.

[80] Wajant H. (2003). Targeting the FLICE Inhibitory Protein (FLIP) in cancer therapy. *Molecular Interventions*.

[81] Geserick P., Drewniok C., Hupe M. (2008). Suppression of cFLIP is sufficient to sensitize human melanoma cells to TRAIL- and CD95L-mediated apoptosis. *Oncogene*.

[82] Couet J., Belanger M. M., Roussel E., Drolet M.-C. (2001). Cell biology of caveolae and caveolin. *Advanced Drug Delivery Reviews*.

[83] Miotti S., Tomassetti A., Facetti I., Sanna E., Berno V., Canevari S. (2005). Simultaneous expression of caveolin-1 and E-cadherin in ovarian carcinoma cells stabilizes adherens junctions through inhibition of src-related kinases. *The American Journal of Pathology*.

[84] Bailey K. M., Liu J. (2008). Caveolin-1 up-regulation during epithelial to mesenchymal transition is mediated by focal adhesion kinase. *The Journal of Biological Chemistry*.

[85] McKie A. B., Vaughan S., Zanini E. (2012). The OPCML tumor suppressor functions as a cell surface repressor-adaptor, negatively regulating receptor tyrosine kinases in epithelial ovarian cancer. *Cancer Discovery*.

[86] Song K. S., Scherer P. E., Tang Z. (1996). Expression of caveolin-3 in skeletal, cardiac, and smooth muscle cells: caveolin-3 is a component of the sarcolemma and co-fractionates with dystrophin and dystrophin-associated glycoproteins. *The Journal of Biological Chemistry*.

[87] Hehlgans S., Cordes N. (2011). Caveolin-1: an essential modulator of cancer cell radio and chemoresistance. *American Journal of Cancer Research*.

[88] Silva W. I., Maldonado H. M., Velázquez G. (2005). Caveolin isoform expression during differentiation of C6 glioma cells. *International Journal of Developmental Neuroscience*.

[89] Burgermeister E., Liscovitch M., Röcken C., Schmid R. M., Ebert M. P. A. (2008). Caveats of caveolin-1 in cancer progression. *Cancer Letters*.

[90] Monaghan-Benson E., Mastick C. C., McKeown-Longo P. J. (2008). A dual role for caveolin-1 in the regulation of fibronectin matrix assembly by uPAR. *Journal of Cell Science*.

[91] Kronstein R., Seebach J., Großklaus S. (2012). Caveolin-1 opens endothelial cell junctions by targeting catenins. *Cardiovascular Research*.

[92] Williams T. M., Lisanti M. P. (2005). Caveolin-1 in oncogenic transformation, cancer, and metastasis. *The American Journal of Physiology—Cell Physiology*.

[93] Wiechen K., Sers C., Agoulnik A. (2001). Down-regulation of caveolin-1, a candidate tumor suppressor gene, in sarcomas. *The American Journal of Pathology*.

[94] Ho C.-C., Kuo S.-H., Huang P.-H., Huang H.-Y., Yang C.-H., Yang P.-C. (2008). Caveolin-1 expression is significantly associated with drug resistance and poor prognosis in advanced non-small cell lung cancer patients treated with gemcitabine-based chemotherapy. *Lung Cancer*.

[95] Bender F. C., Reymond M. A., Bron C., Quest A. F. G. (2000). Caveolin-1 levels are down-regulated in human colon tumors, and ectopic expression of caveolin-1 in colon carcinoma cell lines reduces cell tumorigenicity. *Cancer Research*.

[96] Witkiewicz A. K., Dasgupta A., Sammons S. (2010). Loss of stromal caveolin-1 expression predicts poor clinical outcome in triple negative and basal-like breast cancers. *Cancer Biology & Therapy*.

[97] Karam J. A., Lotan Y., Roehrborn C. G., Ashfaq R., Karakiewicz P. I., Shariat S. F. (2007). Caveolin-1 overexpression is associated with aggressive prostate cancer recurrence. *The Prostate*.

[98] Thompson T. C., Tahir S. A., Li L. (2010). The role of caveolin-1 in prostate cancer: clinical implications. *Prostate Cancer and Prostatic Diseases*.

[99] Ando T., Ishiguro H., Kimura M. (2007). The overexpression of caveolin-1 and caveolin-2 correlates with a poor prognosis and tumor progression in esophageal squamous cell carcinoma. *Oncology Reports*.

[100] Campbell L., Jasani B., Edwards K., Gumbleton M., Griffiths D. F. R. (2008). Combined expression of caveolin-1 and an activated AKT/mTOR pathway predicts reduced disease-free survival in clinically confined renal cell carcinoma. *British Journal of Cancer*.

[101] Fong A., Garcia E., Gwynn L., Lisanti M. P., Fazzari M. J., Li M. (2003). Expression of caveolin-1 and caveolin-2 in urothelial carcinoma of the urinary bladder correlates with tumor grade and squamous differentiation. *American Journal of Clinical Pathology*.

[102] Tanase C. P., Dima S., Mihai M. (2009). Caveolin-1 overexpression correlates with tumour progression markers in pancreatic ductal adenocarcinoma. *Journal of Molecular Histology*.

[103] Nohata N., Hanazawa T., Kikkawa N. (2011). Caveolin-1 mediates tumor cell migration and invasion and its regulation by miR-133a in head and neck squamous cell carcinoma. *International Journal of Oncology*.

[104] Murakami S. M., Miyamoto M., Hida Y. (2003). Caveolin-I overexpression is a favourable prognostic factor for patients with extrahepatic bile duct carcinoma. *British Journal of Cancer*.

[105] Shi L., Chen X.-M., Wang L., Zhang L., Chen Z. (2007). Expression of caveolin-1 in mucoepidermoid carcinoma of the salivary glands: correlation with vascular endothelial growth factor, microvessel density, and clinical outcome. *Cancer*.

[106] Miotti S., Bagnoli M., Tomassetti A., Colnaghi M. I., Canevari S. (2000). Interaction of folate receptor with signaling molecules lyn and G(alpha)(i-3) in detergent-resistant complexes from the ovary carcinoma cell line IGROV1. *Journal of Cell Science*.

[107] Bagnoli M., Tomassetti A., Figini M. (2000). Downmodulation of caveolin-1 expression in human ovarian carcinoma is directly related to alpha-folate receptor overexpression. *Oncogene*.

[108] Bagnoli M., Canevari S., Figini M. (2003). A step further in understanding the biology of the folate receptor in ovarian carcinoma.. *Gynecologic Oncology*.

[109] Prinetti A., Cao T., Illuzzi G. (2011). A glycosphingolipid/caveolin-1 signaling complex inhibits motility of human ovarian carcinoma cells. *The Journal of Biological Chemistry*.

[110] Davidson B., Nesland J. M., Goldberg I. (2001). Caveolin-1 expression in advanced-stage ovarian carcinoma—a clinicopathologic study. *Gynecologic Oncology*.

[111] Wiechen K., Diatchenko L., Agoulnik A. (2001). Caveolin-1 is down-regulated in human ovarian carcinoma and acts as a candidate tumor suppressor gene. *American Journal of Pathology*.

[112] Quest A. F. G., Gutierrez-Pajares J. L., Torres V. A. (2008). Caveolin-1: an ambiguous partner in cell signalling and cancer. *Journal of Cellular and Molecular Medicine*.

[113] Razani B., Wang X. B., Engelman J. A. (2002). Caveolin-2-deficient mice show evidence of severe pulmonary dysfunction without disruption of caveolae. *Molecular and Cellular Biology*.

[114] Pelkmans L., Zerial M. (2005). Kinase-regulated quantal assemblies and kiss-and-run recycling of caveolae. *Nature*.

[115] Carver L. A., Schnitzer J. E. (2003). Caveolae: mining little caves for new cancer targets. *Nature Reviews Cancer*.

[116] Zhang W., Razani B., Altschuler Y. (2000). Caveolin-1 inhibits epidermal growth factor-stimulated lamellipod extension and cell migration in metastatic mammary adenocarcinoma cells (MTLn3). Transformation suppressor effects of adenovirus-mediated gene delivery of caveolin-1. *The Journal of Biological Chemistry*.

[117] Tekpli X., Holme J. A., Sergent O., Lagadic-Gossmann D. (2013). Role for membrane remodeling in cell death: implication for health and disease. *Toxicology*.

[118] Sun J., Gao J., Hu J. B. (2012). Expression of Cav-1 in tumour cells, rather than in stromal tissue, may promote cervical squamous cell carcinoma proliferation, and correlates with high-risk HPV infection. *Oncology Reports*.

[119] Sun L. M., Wang H. Y., Wang Z. G. (2012). Mixed lineage kinase domain-like protein mediates necrosis signaling downstream of RIP3 kinase. *Cell*.

